# Cytokine Signature in Schnitzler Syndrome: Proinflammatory Cytokine Production Associated to Th Suppression

**DOI:** 10.3389/fimmu.2020.588322

**Published:** 2020-11-26

**Authors:** Marie Masson Regnault, Eric Frouin, Isabelle Jéru, Adriana Delwail, Sandrine Charreau, Sébastien Barbarot, Antoine Néel, Agathe Masseau, Xavier Puéchal, Xavier Kyndt, Stephane Gayet, François Lifermann, Bouchra Asli, Xavier Balguerie, Claire Blanchard-Delaunay, François Aubin, Rita Rizzi, Franco Rongioletti, Thierry Boyé, Laurence Gusdorf, Didier Bessis, Franck Morel, Ewa Hainaut, Dan Lipsker, Jean-Claude Lecron

**Affiliations:** ^1^ Centre Hospitalo-Universitaire de Poitiers, Service de Dermatologie, Poitiers, France; ^2^ Laboratoire Inflammation, Tissus Epithéliaux et Cytokines (LITEC), EA4331, Université de Poitiers, Poitiers, France; ^3^ Centre Hospitalo-universitaire, Service de Anatomopathologie, Poitiers, France; ^4^ Sorbonne Université, Inserm UMR 933, Childhood Genetic Disorders, Hôpital Trousseau, Paris, France; ^5^ ImageUP, Plate-forme d’Imagerie et Laboratoire Signalisation et Transport Ioniques Membranaires ERL CNRS 7003/EA 7349, Université de Poitiers, Poitiers, France; ^6^ Centre Hospitalo-universitaire de Nantes, Service de Dermatologie, Nantes, France; ^7^ CHU Nantes, Service de Médecine Interne, Nantes, France; ^8^ CHU Nantes, Université de Nantes, Inserm, Centre de Recherche en Transplantation et Immunologie, UMR 1064, ITUN, Nantes, France; ^9^ Centre de Référence Maladies Systémiques et Auto-Immunes Rares, Université Paris Descartes, APHP, Hôpital Cochin, Paris, France; ^10^ Centre Hospitalier de Valenciennes, Service de Médecine Interne, Valenciennes, France; ^11^ Service de Medecine Interne, Centre hospitalo-Universitaire La Timone, Marseille, France; ^12^ Centre Hospitalier de Dax, Service de Médecine Interne Hématologie, Dax, France; ^13^ Centre Hospitalier Edouard Herriot-Lyon, Service de Médecine Interne, Lyon, France; ^14^ Centre Hospitalier de Rouen, Service de Dermatologie, Rouen, France; ^15^ Centre Hospitalier de Niort, Service de Médecine Interne, Niort, France; ^16^ Centre Hospitalier de Besançon, Service de Dermatologie, Besançon, France; ^17^ Department of Hematology, University of Bari Medical School, Bari, Italy; ^18^ Department of Medical Sciences and Public Health, Unit of Dermatology, University of Cagliari, Cagliari, Italy; ^19^ Service de Dermatologie, Hôpital d’instruction des Armées Sainte-Anne, Toulon, France; ^20^ Centre Hospitalier Universitaire de Reims, Service de Dermatologie et Vénéréologie, Reims, France; ^21^ Centre Hospitalier Universitaire de Montpellier, Hôpital Saint-Eloi, Service de Dermatologie et Vénéréologie, Montpellier, France; ^22^ Faculté de Médecine, Université de Strasbourg et Clinique Dermatologique, Hôpitaux Universitaires de Strasbourg, Strasbourg, France; ^23^ CHU de Poitiers, Laboratoire Immunologie-Inflammation, Poitiers, France

**Keywords:** Schnitzler syndrome, inflammasome, IL-1, IL-1 antagonist, cytokines, PBMC (peripheral blood mononuclear cells), *ex vivo*

## Abstract

**Background:**

Schnitzler syndrome (SchS) is a rare autoinflammatory disease characterized by urticarial exanthema, bone and joint alterations, fever and monoclonal IgM gammopathy. Overactivation of the interleukin(IL)-1 system is reported, even though the exact pathophysiological pathways remain unknown.

**Objective:**

To determine *ex* v*ivo* cytokine profiles of Peripheral Blood Mononuclear Cells (PBMCs) from SchS patients prior to treatment and after initiation of anti-IL-1 therapy (anakinra). The sera cytokine profile was studied in parallel.

**Methods:**

We collected blood samples from thirty-six untreated or treated SchS. PBMCs were cultured with and without LPS or anti-CD3/CD28. Cytokine levels were evaluated in serum and cell culture supernatants using Luminex technology.

**Results:**

Spontaneous TNFα, IL-6, IL-1β, IL-1α, and IL-1RA release by PBMCs of SchS patients were higher than in controls. LPS-stimulation further induced the secretion of these cytokines. In contrast, after T-cell stimulation, TNFα, IL-10, IFNγ, IL-17A, and IL-4 production decreased in SchS patients compared to healthy controls, but less in treated patients. Whereas IL-1β serum level was not detected in most sera, IL-6, IL-10, and TNFα serum levels were higher in patients with SchS and IFNγ and IL-4 levels were lower. Of note, IL-6 decreased after treatment in SchS (*p* = 0.04).

**Conclusion:**

Our data strengthen the hypothesis of myeloid inflammation in SchS, mediated in particular by IL-1β, TNFα, and IL-6, associated with overproduction of the inhibitors IL-1RA and IL-10. In contrast, we observed a loss of Th1, Th2, and Th17 cell functionalities that tends to be reversed by anakinra.

## Introduction 

Schnitzler syndrome (SchS), first described in 1972 by Liliane Schnitzler (1), entered into the classification of autoinflammatory syndromes several decades later, when knowledge of this group of disorders involving the innate immune system started to improve. SchS is characterized by chronic urticarial exanthema with neutrophilic infiltrate, monoclonal gammopathy, and signs of systemic inflammation including recurrent fever attacks, as well as bone and joint manifestations. The paraprotein typically belongs to the IgM or less often to the IgG class (2). Its role, acting as a cause or as a consequence of the inflammatory process, remains a question to be resolved. Systemic inflammation is revealed by elevated levels of C-reactive protein, together with leukocytosis, neutrophilia and/or monocytosis. About 15–20% of patients with Schnitzler syndrome develop lymphoproliferative diseases and, in rare cases, amyloid A (AA) amyloidosis can occur if the disease is not treated (3–5). Besides clinical signs, patients suffer from major quality of life impairment with impact on social and professional life (6). It is a late-onset disease compared to most other autoinflammatory disorders, with median age at clinical onset of 55 years. It affects both males and females with a ratio of 1.5:1 (7). Up until now about 300 cases have been reported throughout the world, most patients coming from Europe, Australia, United States and Japan (8, 9).

The first clue to the autoinflammatory nature of the disease came from the high efficacy of treatment with anakinra, an anti-IL1 receptor antagonist. This drug was tested in light of its efficacy in patients with cryopyrin-associated periodic syndrome (CAPS) and the clinical similarities between SchS and CAPS, a monogenic autoinflammatory disease due to pathogenic variants in the *NLRP3* gene. To date, no specific gene has been implicated in SchS and it is classified as an acquired autoinflammatory disease. Nevertheless, two patients with IgG-type SchS were found to carry variants in *NLRP3*. Notably, in these situations the variants were found only in cells of myeloid lineage, consistent with a somatic mosaicism and raising the question of misdiagnosed CAPS (10). Over the last past 10 years, IL-1 blocking therapies have been proven highly successful in achieving complete control of disease symptoms (11). This suggests a crucial role of IL-1 in disease induction, similar to what is observed in several other autoinflammatory disorders.

Despite increased disease awareness, SchS remains underdiagnosed and associated with a diagnostic delay of several years or even decades. In addition, the exact molecular and cellular mechanisms underlying SchS remain largely unknown. This underlines a crucial need to gain better insight into the disease pathophysiology and to characterize new biomarkers. A number of reports argue for a crucial role of the cytokine IL-1 pathway in the pathogenic process, and the potential role of a few other cytokines has been discussed (12–14). However, additional studies are needed to get a more general view of the cytokine secretion profile characterizing this inflammatory disorder. Studies up until now have been limited and sometimes contradictory and have not yielded a clear-cut SchS cytokine pattern. Since measurement of circulating IL-1β is not a reliable indicator of its fundamental role in systemic inflammatory and autoinflammatory diseases, alternative approaches have been designed by our laboratory and others to assess cytokine status (15). Whole blood or peripheral blood mononuclear cells (PBMCs) cultures may enable study of cytokine production *ex vivo* spontaneously or after activation of specific receptors and/or cells, thereby reflecting their secretory potential in defined condition. By using such an approach, we previously reported overproduction of IL-1β by PBMCs in patients with NLRP3, NLRP12, or MEFV mutations (16–18).

Since most studies related to SchS correspond to case reports, while cohort studies are still lacking, we undertook a prospective study including 36 patients. This is the largest series of patients presenting with SchS studied *ex vivo*. Data obtained on patient PBMCs were compared to cytokine profiles in sera. We also evaluated the impact in five individuals of anti-IL-1 treatments on cytokine signature, in association with clinical benefits.

## Material and Methods

### Ethics

The study was reviewed and approved by a research ethics committee of the University Hospital, Strasbourg, France.

### Patients

We constructed a prospective multicentric study (see https://clinicaltrials.gov/ct2/show/NCT00933296). Patients with SchS syndrome were recruited between April 2010 and July 2013. Thirty-six patients consisting of 22 males and 14 females were included. The subjects enrolled were between 34 and 84 years of age. All had a definite diagnosis of Schnitzler syndrome according to Lipsker and Strasbourg criteria (see Gusdorf et al.). Patients were classified as treated (anakinra) or untreated. Patients were considered treated if they had begun anakinra since at least one week. They were considered untreated after a washout period for more than one week before inclusion (excepted for one patient with 3 days of washout). Of note, Schnitzler disease is a very rare orphan disorder and there is no approved treatment in France. Therefore, treatment relies on expert recommendations. For the patients included in this study, there was no ethical issue, since anakinra is the recommended treatment of Schnitzler (2).

We collected blood samples from of 23 untreated and 19 treated SchS patients for *ex vivo* analyses and from 21 untreated and 15 treated patients for serum collection. For five patients, we collected sera samples both with and without anakinra treatment. Data collection included demographic characteristics. The control group consisted of 21 healthy volunteers for serum level analysis (12 males and 9 females, from 22 to 67 years of age). Among them, 10 healthy volunteers had *ex vivo* analysis (5 males and 5 females from 26 to 56 years of age). The study was in compliance with the declaration of Helsinki. All participants gave their written informed consent.

### Samples

Ten milliliters of blood from patients and healthy controls were collected in Vacutainer tubes containing heparin for the isolation of PBMCs and 3 ml in dry Vacutainer tubes for sera. Sera were obtained by centrifugation and stored at -80°C until analysis.

### Isolation of Peripheral Blood

Human PBMCs were isolated from peripheral heparinized blood by density gradient centrifugation using Ficoll-Paque (GE Healthcare) and counted.

### Cell Culture

The study of spontaneous release informs on the ability of PBMCs to produce cytokines, indicating the presence of activated circulating cells in the disease. We used LPS to further activate monocytes by TLR-4 stimulation and anti-CD3/CD28 to activate T lymphocytes within PBMCs, in order to reveal underlying intrinsic dysfunctions of these cell populations in the disease. To estimate lymphocyte activities, we investigated the pro- and anti-inflammatory cytokines with a set of cytokines representative of Th subsets. Even if they are not exclusively secreted by those Th subsets, IFNγ, IL-4, IL-17, and IL-10 were representative of Th1 cell, Th2 cell, Th17 cell, and Treg cell, respectively (15). Isolated PBMCs were cultured in RPMI 1640 containing 2 mM glutamine, 100 U/mL penicillin-streptomycin (Invitrogen Life Technologies), and 10% heat-inactivated fetal calf serum (Gibco Life Technologies). Cells were seeded at 1 × 10^6^ cells/ml in 24-well plates and incubated 24 h at 37°C in a humidified atmosphere containing 5% CO_2_. Cultures were either left unstimulated or stimulated thorough the 24 h culture period with *E. coli* LPS (Lipopolysaccharide) (1 µg/mL) or anti-CD3/CD28 beads (2.5 × 10^5^ beads/10^6^ cells/ml) (Invitrogen Life Technologies). After incubation, plates were centrifuged and supernatants were stored at -80°C until analysis.

### Cytokine and CRP Measurements

The concentrations of IL-1α, IL-1β, IL-1RA, IL-4, IL-6, TNFα, IL-10, and γ interferon (IFNγ) were quantified in serum and supernatants with the Luminex 200™ plateform (Luminex Xmap Technology) coupled with xPONENT™ software by using the MILLIPLEX MAP Human Cytokine/Chemokine magnetic bead panel kit (Millipore Corporation, Billerica, MA) according to the manufacturer’s instructions. All samples were assayed in duplicates. The concentrations of CRP in serum were quantified by immunoturbidimetry using a Cobas analyser (Roche, Bale, Switzerland)

### Statistical Analysis

All statistical analyses were performed using GraphPad Prism 5 (GraphPad Software, Inc.). Descriptive statistics were presented as median and range (minimum - maximum). Kruskal–Wallis test was conducted to examine the differences between the groups: treated patients, untreated patients and healthy subjects. Then, nonparametric Mann-Whitney U test was used to evaluate the difference between two groups. Those paired were compared with paired t-test. P values less than 0.05 were considered statistically significant for Kuskal-Wallis test, and p values less than 0.025 were considered statistically significant for Mann-Whitney U test after the Bonferroni correction. For statistics, concentrations below the detection limit were considered as half of the limit detection level.

## Results

### Spontaneous and Induced Cytokine Production by PBMCs of SchS Patients

**Figure 1:**
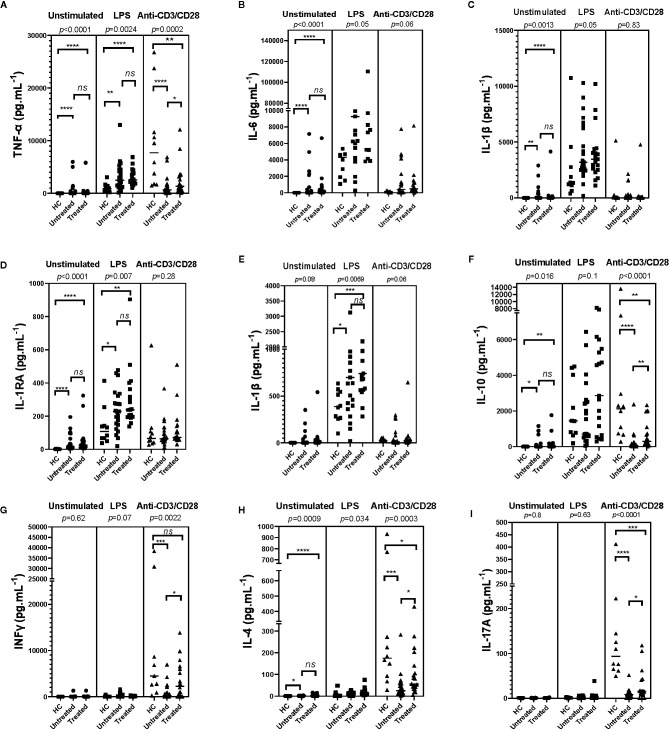
Monocyte/lymphocyte derived-cytokines in supernatants of Schnitzler patients (23 untreated and 19 treated SchS patients) compared to healthy controls (HC) (n = 10). In patients, measurements were made prior to any treatment and/or after initiation of an anti-IL-1 therapy (anakinra). PBMCs were cultured at 1x10^6^ cells/ml for 24 h at 37°C, 5% CO_2_ with and without stimulation of monocytes by *E. coli* LPS (1 µg/mL) or T lymphocytes by anti-CD3/CD28 (2.5 × 10^4^ beads/10^6^ cells/ml). Supernatants were collected and cytokine levels were assayed by Luminex Multiplex Elisa. (Difference between the groups using Kruskal–Wallis test was presented in the top of the figure for each conditions. ****p < 0.0001, ***p < 0.001, **p < 0.01, *p < 0.025 based on nonparametric Mann-Whitney U test for differences between Schnitzler patients and healthy controls). ns, not significant.

TNFa, IL-6, and IL-1 cytokine release: Spontaneous IL-1β, IL-6, TNFα, and IL-1RA release by PBMCs of untreated and anakinra-treated SchS patients were higher than in controls (**Figures 1A–D**). However, these cytokine levels were comparable between untreated and anakinra-treated SchS patients. For IL-1α, spontaneous release by PBMCs was slightly higher in untreated patients than in controls (**Figure 1E**). LPS-stimulated PBMCs from untreated or treated SchS patients produced a significant higher levels of TNFα, IL-1α, IL-1β, and IL-1RA compared to LPS-stimulated PBMCs from healthy controls, whereas IL-6 levels were not significantly increased. Of note, treatment of patients with anakinra does not influence the levels of these cytokines in LPS-stimulated supernatants. In contrast, after T-cell stimulation with anti-CD3/CD28, TNFα production were lower in treated or untreated SchS patients compared to healthy controls, and treated patients had significantly higher TNFα production than untreated patients. No noticeable difference in IL-6, IL-1β, IL-1α, and IL-1RA production was found.

*IL-10* *release:* Spontaneous IL-10 release by PBMCs in untreated and treated patients was higher than in controls, but similar between untreated and treated patients. No difference was found after LPS stimulation. After T-cell stimulation, IL-10 production was lower in treated or untreated SchS patients compared to healthy controls, and treated SchS patients had higher production of IL-10 than untreated patients, as was the case with TNFα production (**Figure 1F**).

*IFNγ release:* IFNγ spontaneous release by PBMCs in patients was not significantly different than in controls, and as anticipated, no difference was found after LPS stimulation. As expected, CD3 and CD28 stimulation induced huge IFNγ production compared to unactivated PBMCs, and IFNγ production was significantly lower in untreated SchS patients compared to healthy controls. Moreover, treated SchS patients had significant higher production of IFNγ than untreated patients, as was the case with TNFα and IL-10 (**Figure 1G**).

*IL-4* *release:* In unstimulated or LPS conditions, IL-4 levels were higher in PBMCs culture supernatants of SchS patients compared to the control group, and no difference was found between untreated or treated SchS patients. For healthy subjects under T-cell stimulation conditions, IL-4 production was strongly enhanced compared to unactivated PBMCs. SchS untreated or anakinra-treated patients had lower IL-4 production than control subjects, and untreated patients had lower IL-4 levels than treated patients. (**Figure 1H**).

*IL-17* *release:* Spontaneously or after LPS stimulation, IL-17A release by PBMCs of SchS patients was not different than in controls. In contrast and as expected, after T-cell stimulation IL-17A production was enhanced compared to unstimulated PBMCs and was lower in treated or untreated SchS patients compared to healthy controls. SchS patients treated had significantly higher production of IL-17A than untreated patients, as also shown for TNFα, IL-10, IFNγ, and IL-4 production (**Figure 1I**).

Taken together, we found high spontaneous production of TNFα, IL-6, IL-1β, and IL-1RA, especially for TNFα and IL-6. We observed decreased IL-4, IL-10, IL17A, TNFα, and IFNγ production by activated T cells of PBMCs from SchS patient, which could be partially restored by anakinra treatment.

### Circulating Levels of Cytokines and CRP in Treated and Untreated Schnitzler Patients

IL-6, IL-10, and TNFα serum levels were higher in the sera of SchS patients, such as CRP levels, compared to healthy controls (*p* < 0.0001; *p* < 0.0001; *p* = 0.01 respectively) (**Table 1**). Of note, IL-6 and CRP decreased significantly after treatment in SchS patients (*p* = 0.008, *p* = 0.001), but no change was noted in TNFα and IL-10. IFNγ and IL-4 levels were lower in sera of SchS patients compared to healthy controls (*p* < 0.0001 and *p* = 0.002, respectively), while IFNγ and IL-4 increased slightly after treatment (*p* = 0.04 and *p* = 0.67, respectively).

**Table 1:** Cytokine levels in sera of patients before and/or after initiation of anakinra, as compared to healthy controls (Mann-Whitney U test).

	Untreated SchS (n = 21)	*p*-value between untreated SchS and controls	Treated SchS (n = 15)	*p*-value between treated SchS and controls	*p*-value between treated and untreated SchS	Healthy controls (n = 21)
**IFNγ (pg/mL)**	<3.2 (<3.2–7.6)	<0.0001	<3.2 (<3.2–9.7)	0.011	0.041	4.28 (<3.2–51.8)
**IL-10 (pg/mL)**	<3.2 (<3.2–41.1)	0.0009	<3.2 (<3.2–17.3)	0.008	0.397	<3.2 (<3.2–9.8)
**IL-17A (pg/mL)**	<3.2 (<3.2–10.8)	0.09	<3.2 (<3.2–19.0)	0.148	0.982	<3.2 (<3.2–25.4)
**IL-1 RA (pg/mL)**	20.2 (<3.2–543.2)	0.021	Non valuable	–	–	7.58 (<3.2–39.5)
**IL-1α (pg/mL)**	<3.2 (<3.2–2.8)	0.277	<3.2 (<3.2–62.5)	0.856	0.382	<3.2 (<3.2–78.8)
**IL-1β (pg/mL)**	<3.2 (<3.2–31.8)	0.944	<3.2 (<3.2–62.)	0.376	0.439	<3.2 (<3.2–18.2)
**IL-4 (pg/mL)**	<3.2 (<3.2–26.3)	0.002	<3.2 (<3.2–60.2)	0.02	0.67	<3.2 (<3.2–55.1)
**IL-6 (pg/mL)**	11.1 (<3.2–177)	<0.0001	<3.2 (<3.2–750)	0.152	0.008	<3.2 (<3.2–12.3)
**TNFα (pg/mL)**	11.7 (1.9–117.0)	0.01	112 (2.5–97.0)	0.02	0.74	6.16 (2.4–14.2)
**CRP (mg/L)**	27 (<2–222)	<0.0001	2.4 (<2–106)	0.02	0.001	<2 (<2–2.8)

Data are presented as medians (min-max).

IL-1-RA increased in SchS patients compared to controls (*p* = 0.02). There was no difference in IL-1α and IL-17 levels between SchS patients and controls.

**Figure 2:**
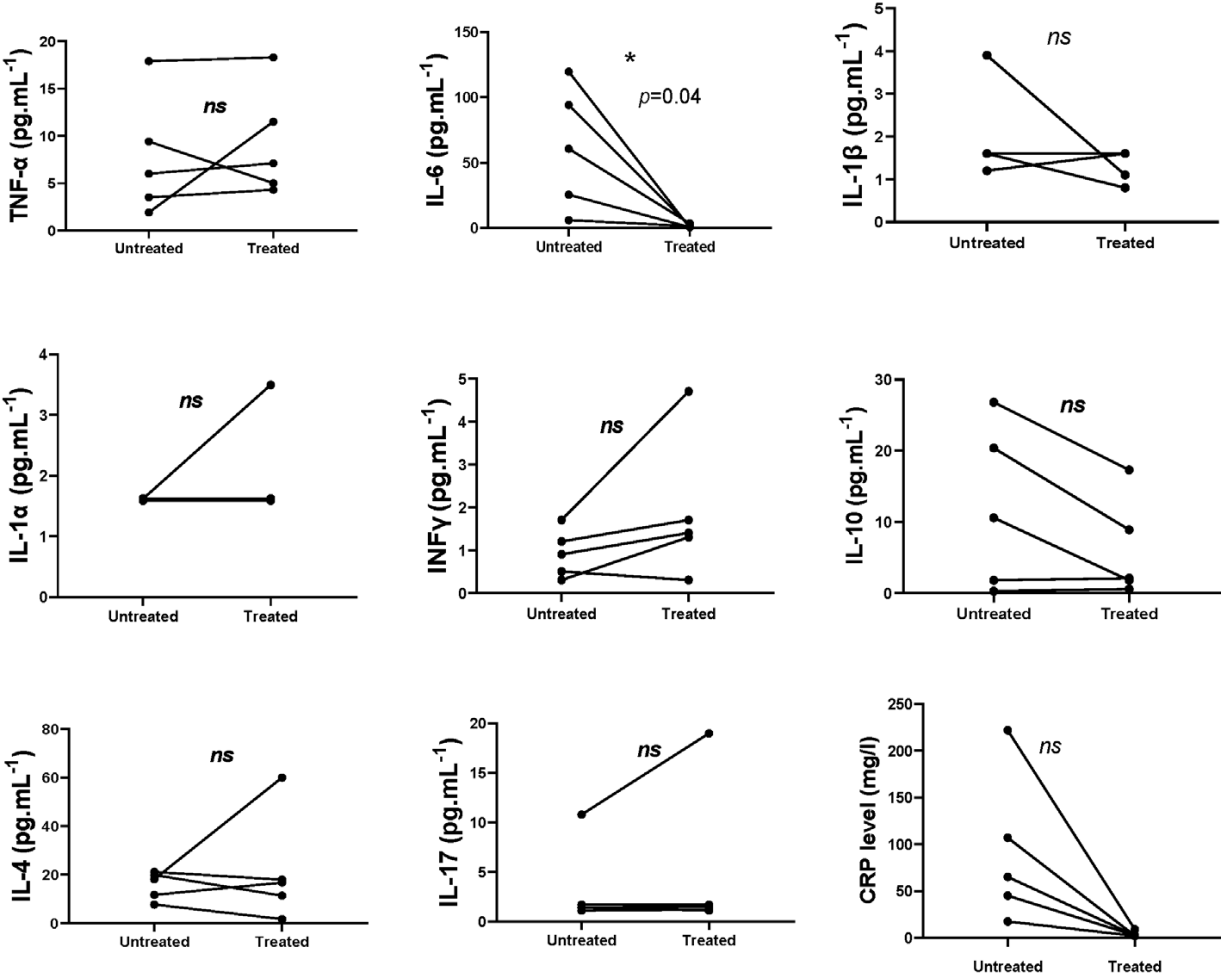
Measurement of cytokine levels in patients pre and post-treatment with anakinra (paired t-test, IL-10 (*p* = 0.08); INFg (*p* = 0.15); IL-17 (*p* = 0.39); IL-1α (*p* = 0.37); IL-1β (*p* = 0.32); IL-6 (*p* = 0.04); IL-4 (*p* = 0.56); TNF (*p* = 0.54); CRP (*p* = 0.06)). ns, not significant. *p < 0.05.

In five patients sera samples were collected before and after anakinra introduction. Data on paired samples are in agreement with the above results. Decreased IL-6 and CRP serum levels after treatment was observed (*p* = 0.04, *p* = 0.06) (**Figure 2**).

## Discussion

The concept of autoinflammation arose twenty years ago from the recognition of monogenic disorders with seemingly unprovoked inflammatory attacks without high-titer autoantibodies or antigen-specific T cells (19). A direct link was then drawn between these autoinflammatory diseases and a dysfunction of the innate immune system, with targeted therapies providing a powerful affirmation of mechanistic hypotheses. Nowadays, the spectrum of autoinflammatory disorders keeps expanding. A new challenge will be to distinguish these different autoinflammatory entities, when they are not monogenic, and to provide the most appropriate treatment to patients. In this regard, cytokine profile signatures could represent useful biomarkers for both diagnosis and evaluation of response to targeted therapy.

In this large prospective series including 36 patients with SchS, we studied a panel of nine cytokines in both sera and PBMCs cultured *ex vivo*. The last approach can be performed on purified lymphocytes and monocytes or PBMCs. Both strategies, each with respective advantages, were reported. Using purified T cells or monocytes allows to individually define the place of each cell population. We have chosen to study PBMCs in order to have a more integrated physiologic response involving relationship between the different cells, an approach also more convenient to implement with a large set of patients. We found elevated spontaneous production of TNFα, IL-6, IL-1β, and IL-1RA by PBMCs, the highest levels being observed for IL-6 and IL-1β. In contrast, the production of IL-4, IL-10, IL17A, TNFα, and IFNγ by activated T cells of PBMCs decreased, and the decrease could be partially reversed by treatment with anakinra. These data provide a new biological signature of SchS. Consistent with our data, Pizzirani et al. reported higher spontaneous production of IL-1β by PBMCs from a SchS patient compared to control (14), whereas three other studies did not observe any differences, including for TNFα and IL-6 (12, 20, 21). Although informative, the major limitation of these works is that they were performed on one or two subjects, whereas we studied a large series of patients. Regarding LPS-stimulated PBMCs, in accordance with our results, Ryan et al. showed that PBMCs from a patient with SchS produced higher IL-1β, TNFα and IL-6 than controls (12). This finding was confirmed by Launay et al., who reported *in vivo* and *in vitro* effects of anakinra on IL-1β and IL-6 production (13, 21). When monocytes were isolated from PBMCs, higher spontaneous production of IL-1β by cells from a SchS patient compared to control, both at a protein and at a mRNA level, was described (12, 21). Interestingly, we also found a slight increase in IL-1α release from LPS-stimulated PBMCs, an observation that may explain why some SchS patients felt better under anakinra, which antagonizes the effects of both IL-1α and IL-1β, than under canakinumab, an anti-IL-1β antibody (D. Lipsker, personal observation). Also consistent with our results on IL-6, several previous studies have reported complete disease remission in a few patients treated with tocilizumab, an anti-IL-6 antibody, indicating that IL-6 also plays a pivotal role in the pathogenesis of SchS (6, 20, 22).

The involvement of adaptive immunity and T lymphocyte sub-populations has been poorly evaluated in SchS patients. In our study, we reported that the production of IL-4, IL-10, IL17A, TNFα, and IFNγ production by activated T cells of PBMCs decreased in treated or untreated SchS patients compared to healthy controls. These results suggest T cell immunosuppression, which applies to Th1, Th2, Th17, and Treg functions. Interestingly, these cytokine productions tend to be restored in anakinra-treated patients, without reaching that of control subjects. This observation suggests that anakinra efficiency could be associated with the restoration of Th functions, even if this hypothesis needs to be studied more in detail. Focusing on Th17 sub-populations, Noster et al. reported that systemic overproduction of IL-1β translates into a loss of anti-inflammatory Th17 cells, characterized by IL-17 production, but not IL-10 (23). After IL-1 blocking therapy, these authors reported restored IL-10 expression and the regulatory properties of Th17. A similar decrease of IL-10 expression has been described in other auto-inflammatory diseases that are known to have increased IL-1β levels, such as systemic juvenile idiopathic arthritis (24). Moreover, a defect in Treg in other chronic inflammatory diseases such as rheumatoid arthritis has also been reported and it was supposed that some drugs may work by promoting the function or increasing numbers of Treg (25). Since we included Th1, Th2, Th17, and Treg by studying the leader cytokines of each of these sub-populations, we found in SchS patients a loss of T cell activities that is not specific to anti-inflammatory Th17 or Treg, and the reversal effect observed in anakinra-treated patients extends to all the cytokines tested. Our data did not find IL-10 synthesis inhibition under anakinra, but rather a restoration of the synthesis. We hypothesize that overproduction of IL-1β in SchS downregulates Th1, Th2, Th17, and Treg together. Using the same approach to study the cytokine signature in FMF, another autoinflammatory disorder, we also reported decreased IFNγ and IL-4 production when compared to controls, whereas IL-10 was unchanged and IL-17 overproduced (16). In a series of eight CAPS patients, an increase of sera IL-17 and Th17 cell number compared to control subjects was reported. Interestingly, Th17 cells decreased after anakinra treatment (26). In accordance, Wilson et al. previously reported an induction of human Th17 cells differentiation induced by IL-1β (27). Of note, NLRP3 inflammasome has been reported to have a crucial role in expansion of Th1/Th17 immunity and in reducing the suppressive control mediated by Treg cells (28). Taken together, our data suggest that this dowregulation of the main T cell subpopulations may be specific to SchS syndrome. Further studies will be required for better understanding of their impact on pathophysiology.

Cytokine-level expression in patient sera is very variable and difficult to interpret because cytokines are immune regulators with short half-lives and their concentrations in serum can be influenced by several variables (15). This is particularly true for IL-1β, which was previously shown to be hardly detectable in autoinflammatory disorders (21). As in other studies, IL-1β serum levels were not detected in 93% of control and SchS samples. In contrast, serum levels of IL-6 and TNFα increased in SchS patients compared to healthy controls, such as CRP. IL-6 and CRP significantly decreased after treatment in SchS patients. Disease activity in Schnitzler syndrome is routinely assessed by clinical evaluation (rash, fever, pain), leukocytosis and CRP level. In accordance with this result, IL-6 levels in serum appeared to be correlated with disease activity in other studies (3, 13). In our study, IFNγ decreased in sera of SchS patients compared to healthy controls and increased after treatment. However, although it was not evaluated in the present study, increased levels of IL-18 [also named interferon-gamma-inducing dactor (IGIF)], an IL-1 cytokine family which plays a major role in the production of IFNγ, have been reported in SchS patients and were reversible with treatment (6, 21, 29). This finding supports the hypothesis that systemic overproduction of IL-1β could lead to a loss in the functionalities of T cells such as Th1, implicated in TNFα and IFNγ production, and Th2, implicated in IL-4 production.

In conclusion, PBMCs in SchS are activated to overproduce IL-1β, consistent with the strong beneficial effect of anti-IL-1 therapies. The elevated levels of the pro-inflammatory cytokines IL-1β, IL-1α, IL-6, and TNFα represent an initial aspect of the cytokine signature in this disorder, similar to that observed in other autoinflammatory disorders. We have to keep in mind that the synergistic induction of TNFα, IL-6, and IL-1 on each of them is a well-known process, which makes it difficult to identify a particular cytokine in the upstream inflammation-inducing process. However, lack of efficacy of TNF-inhibitors in SchS strongly suggests that IL-1 acts upstream. In addition, the T cell cytokine profile, characterized by decreased levels of Th1, Th2, and Th17 cytokines and diminished levels of IL-10 cytokine levels (Treg), represents a specific cytokine ‘‘signature’’ of SchS patients. This new finding about T cell specific dysfunction in patients with SchS syndrome sheds light on unknown aspects of the pathophysiological process and could contribute to the understanding of this complex autoinflammatory disorder.

## Data Availability Statement

The raw data supporting the conclusions of this article will be made available by the authors, without undue reservation.

## Ethics Statement

The studies involving human participants were reviewed and approved by https://clinicaltrials.gov/ct2/show/NCT00933296). The patients/participants provided their written informed consent to participate in this study.

## Author Contributions

MMR, EF, EJ, J-CL, and FM did data analysis and wrote the manuscript. SC and AD did the ex vivo analysis and the cytokines dosages. All the others included patients in the study and have reviewed the paper. All authors contributed to the article and approved the submitted version.

## Conflict of Interest

The authors declare that the research was conducted in the absence of any commercial or financial relationships that could be construed as a potential conflict of interest.

## Acknowledgments

The authors wish to thank Jeffrey Arsham (American medical translator, CHU Poitiers, Poitiers, France) for reviewing and editing the original English language manuscript.

## Abbreviations

SchS, Schnitzler; AA, amyloid A; IL, interleukin; CAPS, cryopyrin-associated periodic; FMF, Familial Mediterranean fever; IFN, interferon; SD, standard deviation; PBMC, peripheral blood mononuclear cells; LPS, lipopolysaccharide; NLRP3, NOD-like Receptor P3; AID, associated autoinflammatory diseases; AMP, antimicrobial peptides.
